# Association between long-term alcohol consumption and insomnia symptoms in civil servants: Aichi Workers’ Cohort Study

**DOI:** 10.20407/fmj.2021-015

**Published:** 2022-01-25

**Authors:** Motoi Terabe, Tsuyoshi Kitajima, Atsuhiko Ota, Hiroshi Yatsuya, Nakao Iwata

**Affiliations:** 1 Department of Psychiatry, Fujita Health University, School of Medicine, Toyoake, Aichi, Japan; 2 Department of Public Health, Fujita Health University, School of Medicine, Toyoake, Aichi, Japan; 3 Department of Public Health and Health Systems, Nagoya University Graduate School of Medicine, Nagoya, Aichi, Japan

**Keywords:** Insomnia, Alcohol consumption, Drinking habits, Alcoholism, Prospective study

## Abstract

**Objectives:**

The influence of habitual alcohol consumption on insomnia symptoms in healthy workers remains unclear. In this study, we evaluated the association between habitual alcohol consumption among civil servants and insomnia symptoms such as difficulty falling asleep, difficulty staying asleep, and tiredness after sleep, using longitudinal data.

**Methods:**

We enrolled civil servants in a prospective cohort study who completed questionnaires at baseline. Of those, 2861 participants were revaluated in a 5-year follow-up survey. Insomnia symptoms during the past month were assessed using self-reporting. Alcohol drinking habits were assessed by querying the frequency of drinking alcohol as well as the amount of alcohol usually consumed per one occasion.

**Results:**

Drinking alcohol every day was less likely to have difficulty falling asleep (odds ratio, 0.42 95% confidence interval, 0.20–0.89), and drinking alcohol 3 or more days a week was associated with difficulty staying asleep (odds ratio, 1.48; 95% confidence interval, 1.16–1.90).

**Conclusions:**

Drinking alcohol every day may produce subjective improvement in sleep onset. However, drinking alcohol 3 or more days a week may increase arousal during sleep, which contributes to reduced sleep quality. These results suggest the possibility that long-term daily habitual drinking may reinforce a sense of improvement in subjective sleep onset but may possibly induce sleep disturbance.

## Introduction

Approximately one in five adults is reported to experience symptoms of insomnia.^1^ The relationship between alcohol consumption and insomnia has been argued and is considered to be complex. It has been reported that ingestion of alcohol causes an acute reduction in sleep onset latency^2^ but disrupts rapid eye movement sleep.^3^ As for the chronic effect of alcohol, continuous drinking to induce sleep may adversely affect sleep quality and intensify the craving for alcohol.^4^ Other evidence shows that heavy drinking of alcohol over an extended period induces tolerance accompanied by adaptation of the neurotransmitter systems.^5,6^ Theoretically, there is a bidirectional relationship between alcohol consumption and sleep disorders but long-term impacts are not yet well understood and remain under investigation.^7,8^

Most previous studies on the association between alcohol consumption and sleep disorders are cross-sectional and have been conducted among patients with alcoholism.^9,10^ To our knowledge, only a few studies have investigated the association between detailed drinking patterns and each symptom of insomnia.^11,12^ The purpose of this study was therefore to evaluate the association between long-term alcohol consumption and each symptom of insomnia in a non-clinical population using longitudinal data.

## Methods

### Design and participants

We conducted a longitudinal study using a dataset obtained from the Aichi Workers’ Cohort Study, a prospective cohort study conducted among civil servants that was initiated in 1997. Follow-up using questionnaire surveys has been carried out every 5 years. Most included civil servants are white-collar workers. About 10% of participants in the present study had shiftwork. We did not exclude workers engaging in shiftwork but performed a sensitivity analysis to confirm that their exclusion did not change the principal findings.

The baseline questionnaire survey used in this study was conducted in 2013 and the follow-up survey was in 2018. Both surveys were conducted from July to August. Of the 5630 civil servants (3889 men) who completed the baseline questionnaire, 2969 continued to work for the local government and participated in the follow-up survey. A total of 2861 participants (2051 men) who did not have insomnia at baseline and who had no missing values for alcohol use, insomnia symptoms, and other covariates were included in our analysis.

This study was approved by the Ethics Review Committee of Nagoya University School of Medicine (Nagoya, Japan) and Fujita Health University (Aichi, Japan), with approval numbers 2013-0005 and HM20-048, respectively. All participants gave their written consent to participate in both the 2013 and 2018 surveys.

### Assessment of insomnia symptoms

In the follow-up survey, we assessed the presence of the following three insomnia symptoms during the previous month: difficulty falling asleep, difficulty staying asleep, and tiredness after sleep. In the assessment, we used participants’ responses to the following three questions: 1) Have you been unable to fall sleep within 30 minutes after going to bed? 2) Have you woken up at night or in the early morning? and 3) Have you felt very tired when you wake up in the morning? All three questions had six possible responses: very rarely; less than once a week; one to two times a week; three to four times a week; five to six a week; every day. The presence of each insomnia symptom was defined as having the symptom more than 3 days a week.^13^

### Assessment of alcohol consumption frequency and amount

Drinking habits were assessed by querying the frequency of drinking alcohol as well as on the amount of alcohol typically consumed on each occasion.

The frequency of drinking was assessed according to six possible responses: rarely; one to three times a month; one to two times a week; three to four times a week; five to six times a week; and every day. In the analysis, the following two cutoff points were used: three times a week or more and every day. These cutoffs were according to the National Health and Nutrition Survey conducted by the Ministry of Health, Labour and Welfare, which defined habitual drinkers as those who consume alcohol at least 3 days per week.^14^

The amount of alcohol consumption per day was assessed in the present study. Participants reported their usual alcohol intake pattern in a day by indicating the number of the following alcoholic beverages (or combinations) consumed: Japanese sake; Japanese distilled spirits; beer; whiskey; wine; and other (respondents were requested to specify the type of alcoholic beverage). The amount of alcohol consumption was calculated as ethanol intake per day. Although use of the average daily intake might be common, the estimate in the present study was alcohol intake per day on days that alcohol was consumed. In the analysis, we used the following two cutoff points: 20 g/day or more and 60 g/day or more. These amounts are defined as average drinking (<20 g/day) and heavy drinking (≥60 g/day) in the Japanese drinking guideline established by the Ministry of Health, Labour and Welfare.^15^

Long-term stable drinking was defined as alcohol consumption at the same level or more in terms of frequency or volume over the 5-year survey interval. For example, if a participants reported drinking every day in both the 2013 and 2018 surveys, we considered that the individual engaged in long-term drinking every day throughout the 5-year study period. The same approach was used for the amount of alcohol consumed; if 60 g/day or more was reported in both surveys, we defined this as long-term drinking of 60 g/day or more.

### Other variables

Previous studies have reported that sex, age, smoking, medical conditions, marital status, and loneliness are associated with individual sleep problems.^16–22^ To control for the possible effect of confounding variables on the association between alcohol drinking and insomnia symptoms, we included the following variables (obtained from the questionnaire) in the model: age, sex, smoking status, sleep apnea syndrome, symptoms of depression, living arrangements (living alone or not), and physical disorders (gastric cancer, bowel cancer, lung cancer, liver cancer, breast cancer, prostate cancer, uterine cancer, stroke, myocardial infarction, angina pectoris, heart failure, atrial fibrillation, hypertension, aortic dissection, aortic aneurysm, diabetes, dyslipidemia, gout, asthma, chronic obstructive pulmonary disease, chronic bronchitis, pulmonary embolism, chronic kidney failure, cataract, glaucoma, age-related macular degeneration, gastrointestinal tract polyps/ulcer, chronic hepatitis, liver cirrhosis, cholelithiasis, ureteral lithiasis, dementia, bone fractures, osteoporosis, and other). Use of sleeping medications was not included as a control variable because we included symptoms of depression defined as a score of 8 or higher in a modified 11-item Center for Epidemiological Studies Depression (CES-D) Scale as a covariate. Nevertheless, we conducted a sensitivity analysis, excluding participants who reported the use of sleeping medications in 2018.

### Statistical analysis

A chi-square test was performed to evaluate the characteristics of participants. We used logistic regression models of each insomnia symptom at follow-up as outcome variables. The explanatory variables were alcohol drinking frequency and amount. In the primary analysis, we examined the association between long-term drinking habits that persisted for 5 years and insomnia symptoms. In the exploratory analysis, we examined the relationship between increased or decreased frequency of alcohol consumption or amount of alcohol consumed and insomnia symptoms during the study period. We determined odds ratios (ORs) and 95% confidence intervals (CIs) using a multivariable-adjusted model that included sex, continuous age, current smoking, living arrangements (cohabitation), medical history, and symptoms of depression. We used IBM SPSS for Windows, version 24 (IBM Corp., Armonk, NY, USA) for the analysis.

## Results

The characteristics of study participants in 2018, when insomnia symptoms were observed, are shown in Table 1. The mean age of the total 2861 participants was 47.4 years, most participants were men (71.7%), and approximately half (50.8%) of participants had a history of physical illness. In 2018, the number of participants with difficulty falling asleep was 244 (8.52%), and 38.8% reported difficulty staying asleep. As for drinking habits over the past 5 years, 387 (13.5%) participants reported drinking every day and 911 (31.8%) drank 3 or more days per week. Nearly all participants who drank alcohol every day were men (92.5%), and the mean age of participants who drank daily was approximately 5 years older than the mean age of those who did not drink every day.

In the primary analysis, participants who consumed alcohol every day (which was assumed to have persisted over the 5-year follow-up period) were less likely to have difficulty falling asleep than participants who did not drink daily (OR, 0.42; 95% CI, 0.20–0.89) independent of the amount of alcohol consumed (Table 2). Alcohol consumption 3 or more days a week was associated with difficulty staying asleep (OR, 1.48; 95% CI, 1.16–1.90), independent of alcohol consumption amount. Drinking alcohol every day was not associated with difficulty staying asleep, and drinking 3 or more days a week was not associated with easily falling asleep. The amount of alcohol consumed was not associated with symptoms of insomnia (Table 3). In the sensitivity analysis, an inverse association between drinking every day and difficulty falling asleep was similar after excluding participants who were taking sleeping medication or who had shiftwork (Tables S1 and S2).

In the exploratory analysis, participants who did not drink alcohol every day in 2013 and reported drinking every day in 2018 were more likely to have difficulty falling asleep than those who did not drink alcohol over the 5-year study period (OR, 1.98; 95% CI, 1.03–3.79), independent of the amount of alcohol consumed (Table S3). Participants who reported consuming 20 g/day or more of alcohol in 2013 but who reported reduced alcohol consumption in 2018 showed an associated with tiredness after sleep (OR, 1.80; 95% CI, 1.17–2.76).

## Discussion

In the primary analysis, we found that compared with drinking alcohol less frequently, drinking every day was inversely associated with difficulty falling asleep, but this association was not found for drinking 3 or more days per week. In contrast, alcohol consumption 3 or more days a week was associated with difficulty staying asleep; however, this association was not observed for drinking every day. We did not observe any meaningful associations between the amount of alcohol consumed and symptoms of insomnia. These findings were not anticipated, as continuously drinking a large amount of alcohol is theoretically associated with disturbed sleep because of tolerance^23^; however, we consider that these apparently discrepant findings could also have important implications.

Although it might be assumed that the results for drinking frequency and amount would be similar, significant associations with sleep status were found for drinking frequency but not for the amount of alcohol consumed. A possible reason for this is that our definition of the amount of drinking in this study was the daily amount of drinking and not the average value converted to a daily basis. Therefore, in theory, more frequent drinking does not necessarily mean more alcohol consumed in 1 day of drinking. Although there was a significant correlation between the frequency of drinking and amount consumed per day, a very small percentage (2%) of the participants met both conditions of drinking every day and drinking 60 g/day persistently.

There are few known longitudinal studies conducted among generally healthy individuals to compare with our findings because most previous studies have included alcohol-dependent individuals, with a primary focus on the volume of alcohol consumption. A recent study by Britton et al. among 10,308 adults in London found that consuming large amounts of alcohol was associated with the prevalence of sleep problems, such as difficulty staying asleep and waking up feeling tired.^11^ Unlike Britton’s study, we found no association between the amount of alcohol consumed and symptoms of insomnia. This discrepancy may be the result of sleep variable dichotomization: Britton et al. assigned different cutoff points to each insomnia symptom because participants were analyzed in groups as similar in size as possible. We did not use the same method in setting the cutoff points. Other possible explanations might be the differences in participants’ age and length of the follow-up period.

At first glance, our results indicate that the continued daily alcohol consumption seems to be a “better” habit for improving difficulty falling asleep than continued drinking on 3 or more days per week. Our findings also seem to indicate that the continuation of drinking alcohol 3 or more days per week may cause an increase in arousal during sleep, but that continued daily alcohol drinking may not. However, drinking 3 or more days per week does not necessarily imply drinking alcohol every day, which may potentially reflect the results regarding sleep arousal on non-drinking days. If this is the case, daily habitual drinking over 5 years would involve some degree of potential risk in terms of being unaware of poor sleep quality; it is possible that dependence would have formed but remains concealed owing to subjective sleep improvement. Continued excessive alcohol intake and repeated withdrawal symptoms have been indicated to contribute to development of alcohol dependence.^24^ Although the participants in our study were not necessarily excessive drinkers, we cannot ignore the possibility that a similar phenomenon might exist to a mild degree. In addition to this, considering the results of previous studies that insomnia causes a risk of alcohol dependence,^9,10,23^ we must consider the possibility that enhanced continuation of alcohol consumption owing to subjective sleep improvement contributes to the development of alcohol dependence.

The results of this exploratory analysis indicated that awareness about the difficulty in falling asleep was significantly more prevalent among participants who did not drink every day in 2013 but who reported drinking every day in 2018. This may suggest that such awareness might have contributed to increasing the frequency of drinking as a sleep aid (Table S3). Another finding in this exploratory analysis was that among participants who drank 20 g/day or more in 2013 and drank a lower amount in 2018, the prevalence of tiredness after sleep was higher than that in respondents who did not drink 20 g/day continuously between 2013 and 2018. Further studies should be conducted to infer possible causal relationships.

We conducted a sensitivity analysis that excluded participants taking sleeping medications or who had shiftwork in 2018, which yielded similar results. This suggests that the influence of these factors on the results was small (Tables S1 and S2).

Our study has several limitations. First, we did not strictly diagnose alcohol dependence or insomnia individually among participants. As for insomnia symptoms, we did not evaluate whether participants had difficulty falling asleep again after arousal, although this has been identified as an important clinical symptom; however, most studies using questionnaires have not assessed this.^12,16,22,25^ The same is true for the Pittsburgh Sleep Quality Index^26^ and Insomnia Severity Index.^27^ Second, we assessed alcohol consumption and sleep using self-reporting; therefore, information biases may be present. Third, we were unable to evaluate weekday and weekend variability and weekly variability in drinking and insomnia using the categorical response choices to the questions used to evaluate alcohol consumption and sleep. Finally, because our data were confined to civil servants, the present findings may not be generalized to other types of employee or to the general population.

Despite these limitations, we identified an association between sleep problems and drinking in a manner that may lead to alcoholism among a large sample of generally healthy individuals. Further research may be required regarding the potential for long-term habitual drinking and the development of dependence associated with insomnia.

## Figures and Tables

**Table1:** Characteristics of participants at follow-up (2018)

	Follow-up	Presence of insomnia symptoms				Persistence of drinking habits throughout 5 years			
	Total subjects N=2861	Difficulty falling asleep N=244 (8.52%)	p	Difficulty stayng asleep N=1109 (38.8%)	p	every day N=387 (13.5%)	p	3 or more days per week N=911 (31.8%)	p
	%	%		%		%		%	
Sex (Men)	71.7	70.1	0.56	74.9	0.002	92.5	<0.001	87.5	<0.001
Age									
Mean, y (SD)	47.4 (9.40)	46.2 (10.1)		49.2 (8.93)		51.7 (6.92)		50.8 (7.64)	
above 60 y/o	6.1	6.1	0.964	8.3	<0.001	9.3	0.012	9.0	<0.001
Smoking +	9.2	11.5	0.273	10.2	0.322	20.2	<0.001	15.1	<0.001
Medical histories									
Physical disorders +	50.8	58.6	0.052	57.3	<0.001	60.0	0.007	57.1	0.01
Sleep Apnea Syndrome +	1.9	3.7	0.041	2.8	0.01	2.1	0.694	2.4	0.227
Symptoms of Depression +	25.2	58.2	<0.001	34.7	<0.001	20.2	0.007	21.1	<0.001
Living arrangements (alone)	10.5	18.4	<0.001	11.2	0.706	6.5	0.01	7.6	<0.001

P-values obtained using chi-square tests, comparing each item with and without insomnia symptom or persistence of drinking habits over the 5-year study period. SD, standard deviation.

**Table2:** Longitudinal association between persistent drinking frequency and insomnia symptoms

		Drinking frequencies			
		Every day –	Every day + OR (95% CI)	3 or more days/week –	3 or more days/week + OR (95% CI)
*Diffuculty falling asleep*	(N=+/–)	N=118/2147	N=8/353	N=83/1693	N=43/870
Model 1 (adjusted with all demographic variables)		*Ref*	0.42 (0.20–0.89)	*Ref*	1.26 (0.84–1.89)
Model 2 (Model 1+alcohol amount, 20 g/day)		*Ref*	0.45 (0.21–0.96)	*Ref*	1.48 (0.95–2.29)
Model 3 (Model 1+alcohol amount, 60 g/day)		*Ref*	0.42 (0.20–0.88)	*Ref*	1.26 (0.84–1.89)
*Diffuculty staying asleep*	(N=+/–)	N=373/1280	N=75/156	N=287/1044	N=148/392
Model 1 (adjusted with all demographic variables)		*Ref*	1.30 (0.93–1.83)	*Ref*	1.48 (1.16–1.90)
Model 2 (Model 1+alcohol amount, 20 g/day)		*Ref*	1.21 (0.85–1.73)	*Ref*	1.44 (1.11–1.87)
Model 3 (Model 1+alcohol amount, 60 g/day)		*Ref*	1.28 (0.80–1.97)	*Ref*	1.47 (1.15–1.89)
*Tiredness after sleep*	(N=+/–)	N=164/1412	N=67/669	N=202/1813	N=29/298
Model 1 (adjusted with all demographic variables)		*Ref*	1.10 (0.71–1.71)	*Ref*	1.04 (0.75–1.44)
Model 2 (Model 1+alcohol amount, 20 g/day)		*Ref*	1.05 (0.67–1.66)	*Ref*	0.99 (0.70–1.40)
Model 3 (Model 1+alcohol amount, 60 g/day)		*Ref*	1.13 (0.73–1.75)	*Ref*	1.05 (0.76–1.46)

Odds ratio (OR) and 95% confidence interval (CI) in logistic regression models. Demographic variables included sex, age (60 years and older), current smoker (yes/no), past medical history of physical disorders, sleep apnea syndrome, symptoms of depression (presence/absence), and living arrangements (living alone or not). Alcohol consumption was 20 g/day (+/–) in model 2 and 60 g/day (+/−) in model 3. Ref, reference category.

**Table3:** Longitudinal association between persistent amount of alcohol consumed and insomnia symptoms

		Alcohol amounts			
		20 g/day –	20 g/day + OR (95% CI)	60 g/day –	60 g/day – OR (95% CI)
*Diffuculty falling asleep*	(N=+/–)	86/1591	40/909	118/2356	8/144
Model 1 (adjusted with all demographic variables)		*Ref*	0.76 (0.51–1.14)	*Ref*	1.03 (0.48–2.19)
Model 4 (Model 1+drinking alcohol everyday)		*Ref*	0.87 (0.58–1.32)	*Ref*	1.14 (0.53–2.44)
Model 5 (Model 1+drinking alcohol 3 or more days/week)		*Ref*	0.66 (0.43–1.02)	*Ref*	0.99 (0.46–2.12)
*Diffuculty staying asleep*	(N=+/–)	271/956	164/480	405/1361	30/75
Model 1 (adjusted with all demographic variables)		*Ref*	1.22 (0.97–1.54)	*Ref*	1.34 (0.87–2.08)
Model 4 (Model 1+drinking alcohol everyday)		*Ref*	1.18 (0.93–1.50)	*Ref*	1.26 (0.80–1.97)
Model 5 (Model 1+drinking alcohol 3 or more days/week)		*Ref*	1.10 (0.86–1.40)	*Ref*	1.24 (0.79–1.94)
*Tiredness after sleep*	(N=+/–)	148/1336	83/775	221/1981	10/130
Model 1 (adjusted with all demographic variables)		*Ref*	1.13 (0.83–1.52)	*Ref*	0.70 (0.36–1.40)
Model 4 (Model 1+drinking alcohol everyday)		*Ref*	1.12 (0.81–1.53)	*Ref*	0.69 (0.35–1.38)
Model 5 (Model 1+drinking alcohol 3 or more days/week)		*Ref*	1.13 (0.82–1.56)	*Ref*	0.70 (0.35–1.39)

Odds ratio (OR) and 95% confidence interval (CI) in logistic regression models. Demographic variables are sex, age (60 years and older), current smoker (yes/no), past medical history of physical disorders, sleep apnea syndrome, symptoms of depression (presence/absence), and living arrangements (living alone or not). Drinking frequency was every day (+/−) in model 4, and 3 or more days (+/−) in model 5. Ref, reference category.
