# Supplementary material for: Association between long-term alcohol consumption and insomnia symptoms in civil servants: Aichi Workers’ Cohort Study
Source: Fujita Med J. 2022 Jan 25;8(4):103–7. doi: 10.20407/fmj.2021-015 (PMC9673082; doi:10.20407/fmj.2021-015)
Supplement: Supplementary file 1 — Supplementary Tables [file fmj-8-103_s001.pdf]

Table S1. A sensitivity analysis excluding the subjects with sleeping medications in 2018. Odds ratios (OR) and their 95% confidence intervals (CI) from logistic regression model (adjusted with all demographic variables)

	Diffuculty falling asleep		Diffuculty staying asleep		Tiredness after sleep	
Every day	N (with/without)	OR (95% CI)	N (with/without)	OR (95% CI)	N (with/without)	OR (95% CI)
Every day -	93/2091	*Ref*	356/1242	*Ref*	191/1758	*Ref*
Every day +	6/332	0.38 (0.16–0.89)	52/149	1.33 (0.94–1.88)	27/280	1.08 (0.69–1.70)
3 or more days						
3 or more days -	66/1644	*Ref*	269/1012	*Ref*	155/1368	*Ref*
3 or more days +	33/779	1.15 (0.74–1.80)	139/379	1.52 (1.18–1.95)	63/670	1.07 (0.76–1.50)
20 g/day						
20 g/day -	67/1543	*Ref*	251/925	*Ref*	137/1291	*Ref*
20 g/day +	32/880	0.76 (0.49–1.18)	157/466	1.27 (1.00–1.60)	81/747	1.10 (0.81–1.51)
60 g/day						
60 g/day -	94/2281	*Ref*	380/1317	*Ref*	209/1909	*Ref*
60 g/day +	5/142	0.74 (0.29–1.88)	28/74	1.27 (0.80–2.01)	9/138	0.65 (0.31–1.32)

Demographic variables are sex, age (60 years and older), current smoker (yes/no), past medical history of physical disorders, sleep apnea syndrome, symptom of depression (presence/absence), and living alone (yes/no).

Table S2. A sensitivity analysis excluding the subjects with shiftwork in 2018. Odds ratios (OR) and their 95% confidence intervals (CI) from logistic regression model (adjusted with all demographic variables)

	Diffuculty falling asleep		Diffuculty staying asleep		Tiredness after sleep	
Every day	N (with/without)	OR (95% CI)	N (with/without)	OR (95% CI)	N (with/without)	OR (95% CI)
Every day -	102/1939	*Ref*	334/1146	*Ref*	177/1639	*Ref*
Every day +	8/334	0.48 (0.23–1.03)	54/147	1.36 (0.96–1.94)	27/287	1.10 (0.70–1.74)
3 or more days						
3 or more days -	71/1517	*Ref*	254/925	*Ref*	143/1268	*Ref*
3 or more days +	39/756	1.30 (0.84–2.00)	134/368	1.45 (1.12–1.88)	61/658	1.04 (0.74–1.46)
20 g/day						
20 g/day -	71/1432	*Ref*	241/850	*Ref*	129/1202	*Ref*
20 g/day +	39/841	0.93 (0.61–1.42)	147/443	1.19 (0.93–1.52)	75/724	1.07 (0.78–1.48)
60 g/day						
60 g/day -	102/2138	*Ref*	361/1222	*Ref*	194/1801	*Ref*
60 g/day +	8/135	1.17 (0.54–2.50)	27/71	1.27 (1.24–0.77)	10/125	0.72 (0.36–1.45)

Demographic variables are sex, age (60 years and older), current smoker (yes/no), past medical history of physical disorders, sleep apnea syndrome, symptom of depression (presence/absence), and living alone (yes/no).

Table S3. An exploratory analysis of the association between increasing and decreacing the frequency or amount of alcohol consumption and insomnia symptoms. Odds ratios (OR) and their 95% confidence intervals (CI) from logistic regression model 1 (adjusted with all demographic variables)

		Diffuculty falling asleep		Diffuculty staying asleep		Tiredness after sleep	
Every day		N (with insomnia symptom)	OR (95% CI)	N (with insomnia symptom)	OR (95% CI)	N (with insomnia symptom)	OR (95% CI)
	kept going not every day	2006 (98)	*Ref*	1483 (333)	*Ref*	1786 (177)	*Ref*
	increased	145 (12)	1.98 (1.03–3.79)	101 (25)	1.16 (0.72–1.87)	131 (14)	1.34 (0.73–2.46)
	decreased	114 (8)	1.55 (0.71–3.37)	74 (20)	1.29 (0.75–2.23)	98 (11)	1.37 (0.70–2.71)
	kept going every day	361 (8)	0.46 (0.22–0.98)	213 (57)	1.34 (0.95–1.88)	327 (29)	1.15 (0.74–1.80)
3 or more days							
	kept going less tha 3 days	1514 (75)	*Ref*	1135 (236)	*Ref*	1333 (135)	*Ref*
	increased	116 (3)	0.64 (0.19–2.09)	90 (22)	1.29 (0.77–2.15)	114 (15)	1.66 (0.91–3.04)
	decreased	146 (5)	0.71 (0.28–1.81)	106 (29)	1.49 (0.94–2.35)	129 (14)	1.19 (0.65–2.19)
	kept going 3 or more days	850 (34)	1.20 (0.79–1.81)	540 (148)	1.56 (1.22–2.03)	766 (67)	1.10 (0.78–1.54)
20 g/day							
	kept going less than 20 g/da	1111 (56)	*Ref*	823 (168)	*Ref*	985 (87)	*Ref*
	increased	226 (17)	1.30 (0.73–2.32)	180 (45)	1.34 (0.91–1.96)	228 (23)	1.36 (0.82–2.26)
	decreased	300 (13)	0.86 (0.46–1.61)	224 (58)	1.40 (0.99–1.98)	271 (38)	1.80 (1.17–2.76)
	kept going more than 20 g/d	949 (40)	0.81 (0.52–1.25)	644 (164)	1.36 (1.06–1.76)	858 (83)	1.28 (0.91–1.79)
60 g/day							
	kept going less than 60 g/da	2184 (102)	*Ref*	1555 (352)	*Ref*	1928 (192)	*Ref*
	increased	156 (11)	1.42 (0.72–2.81)	114 (26)	0.99 (0.59–1.50)	144 (15)	1.10 (0.61–1.99)
	decreased	134 (5)	0.79 (0.31–2.03)	97 (27)	1.35 (0.84–2.16)	130 (14)	1.20 (0.66–2.21)
	kept going more than 60 g/d	152 (8)	1.05 (0.49–2.25)	105 (30)	1.32 (0.84–2.07)	140 (10)	0.72 (0.36–1.43)

Demographic variables are sex, age (60 years and older), current smoker (yes/no), past medical history of physical disorders, sleep apnea syndrome, symptom of depression (presence/absence), and living alone (yes/no).
